# Mechanosensory encoding of forces in walking uphill and downhill: force feedback can stabilize leg movements in stick insects

**DOI:** 10.1152/jn.00414.2023

**Published:** 2024-01-03

**Authors:** Sasha N. Zill, Chris J. Dallmann, William Zyhowski, Hibba Chaudhry, Corinna Gebehart, Nicholas S. Szczecinski

**Affiliations:** ^1^Department of Biomedical Sciences, Joan C. Edwards School of Medicine, Marshall University, Huntington, West Virginia, United States; ^2^Department of Neurobiology and Genetics, Julius-Maximilians-Universität-Würzburg, Würzburg, Germany; ^3^Department of Mechanical and Aerospace Engineering, West Virginia University, Morgantown, West Virginia, United States; ^4^Champalimaud Foundation, Champalimaud Research, Lisbon, Portugal; ^5^Department of Animal Physiology, Institute of Zoology, Biocenter Cologne, University of Cologne, Cologne, Germany

**Keywords:** campaniform sensilla, downhill, force encoding, uphill, walking

## Abstract

Force feedback could be valuable in adapting walking to diverse terrains, but the effects of changes in substrate inclination on discharges of sensory receptors that encode forces have rarely been examined. In insects, force feedback is provided by campaniform sensilla, mechanoreceptors that monitor forces as cuticular strains. We neurographically recorded responses of stick insect tibial campaniform sensilla to “naturalistic” forces (joint torques) that occur at the hind leg femur-tibia (FT) joint in uphill, downhill, and level walking. The FT joint torques, obtained in a previous study that used inverse dynamics to analyze data from freely moving stick insects, are quite variable during level walking (including changes in sign) but are larger in magnitude and more consistent when traversing sloped surfaces. Similar to vertebrates, insects used predominantly extension torque in propulsion on uphill slopes and flexion torques to brake forward motion when going downhill. Sensory discharges to joint torques reflected the torque direction but, unexpectedly, often occurred as multiple bursts that encoded the rate of change of positive forces (dF/d*t*) even when force levels were high. All discharges also showed hysteresis (history dependence), as firing substantially decreased or ceased during transient force decrements. These findings have been tested in simulation in a mathematical model of the sensilla (Szczecinski NS, Dallmann CJ, Quinn RD, Zill SN. *Bioinspir Biomim* 16: 065001, 2021) that accurately reproduced the biological data. Our results suggest the hypothesis that sensory feedback from the femoro-tibial joint indicating force dynamics (dF/d*t*) can be used to counter the instability in traversing sloped surfaces in animals and, potentially, in walking machines.

**NEW & NOTEWORTHY** Discharges of sensory receptors (campaniform sensilla) in the hind legs of stick insects can differentially signal forces that occur in walking uphill versus walking downhill. Unexpectedly, sensory firing most closely reflects the rate of change of force (dF/d*t*) even when the force levels are high. These signals have been replicated in a mathematical model of the receptors and could be used to stabilize leg movements both in the animal and in a walking robot.

## Graphical abstract

**Figure:**
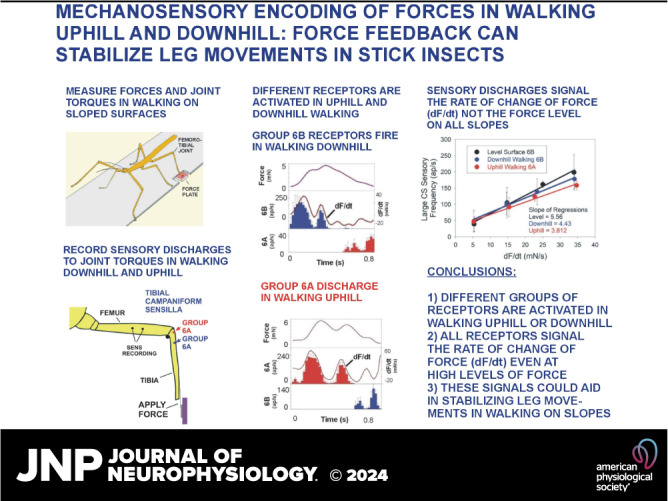

## INTRODUCTION

Walking on sloped surfaces, the natural habitat for most terrestrial animals, requires adaptation of muscle activities to changes in the effects of gravity. The adaptive changes in motor activities in walking on slopes were first precisely and elegantly characterized in cats (Refs. 1–3; comparable studies in humans: Refs. 4–6). In walking on uphill slopes, activities of extensor muscles at a number of leg joints are enhanced to push the animals upward and forward. In traversing downhill slopes, the effects “reverse” and flexor muscle activities are increased and operate in an eccentric mode to brake forward motion during flexor muscle lengthening.

Previous studies have also suggested that receptors monitoring forces in the legs contribute to these adaptations, potentially in diverse ways. Recordings of muscle activities at the ankle in humans walking on a treadmill that could be tilted showed that the extensor muscle activities late in the stance phase of the step cycle were modulated by afferents that detected forces, potentially by a mechanism of positive feedback (7). Modeling and experimental studies in cats walking on pegs in sloped walkways also suggested that Golgi tendon organs (which detect muscle forces and loads) were the primary source of sensory feedback affecting motor outputs at the ankle joint (8). However, studies that have examined multiple joints and intermuscular effects of force receptors have suggested that inhibitory feedback at some leg joints may also play a role in regulating whole limb mechanics (9). Thus, force feedback can have excitatory and inhibitory components that “coexist in various combinations based on motor task” (10).

Studies in stick insects walking on sloped surfaces have shown motor adaptations remarkably similar to those found in vertebrates (Refs. 11–13, review in Ref. 14). Stick insects can ascend or descend nonhorizontal substrates without major changes in the angular movements of most leg joints (13). In contrast, measurements from force plates and recordings of activities in hind leg muscles in freely moving stick insects have shown extensive, joint-specific adaptations in walking on sloped surfaces: at the knee [femoro-tibial (FT) joint] animals generate extensor forces that push the center of mass forward in uphill walking but exert braking (flexor) forces when traversing downhill slopes (13). Comparable changes are seen in muscles at the junction of the leg and body (protractors-retractors), but activities at the intermediate coxo-trochanteral (CTr) joint, which maintains body height above the substrate, were similar on all substrate slopes. The basic similarities in motor strategies in diverse animals most likely reflect solutions to the common biomechanical problems of adapting legged locomotion to changes in the effects of the gravitational vector.

Few studies have directly examined activities of sense organs that could detect these changes in forces in sloped walking in freely moving animals. Recordings of tendon organ afferents in cats walking freely on horizontal surfaces indicate that they signal “a dynamic, non-linear function of whole muscle force over a range encompassing movements involving very low to very high force levels” (15, 16). Modeling studies suggest that force levels can be calculated in the central nervous system (CNS) by summing the ensemble discharge of tendon organs (17), but this has not been confirmed by data from walking animals. In contrast, earlier studies suggest that the dynamic sensitivities of Golgi tendon organs should predominate during walking in some muscles (18) and calculation of force levels would require the temporal integration of sensory signals over time in the central nervous system.

In insects, forces generated by and acting upon the legs are monitored by campaniform sensilla (CS) (19). Activities of tibial campaniform sensilla recorded in freely moving cockroaches show considerable dynamic sensitivities, although forces were not directly measured in those studies (20, 21). Responses of tibial sensilla in the middle legs of stick insects were studied with forces that replicated joint torques of freely moving animals walking on level surfaces and suggested that receptors strongly encode force dynamics (22, 23). However, it was not clear how the receptors would discharge to higher levels of forces necessary in traversing sloped surfaces.

In the present experiments, we have extended the previous studies to examine sensory encoding of joint torques in walking up- and downhill. We examined the encoding of mean torque values and also individual steps, which reflected changes in forces that can occur because of variations in gait in freely moving animals. These studies suggest that sensory discharges of tibial receptors do not simply encode the force level but more closely reflect variations and force dynamics. These signals could be used to compensate for load variations and aid in generating the smooth and continuous joint movements seen in both level and sloped walking.

We also previously developed a mathematical model that captured the response properties of campaniform sensilla (24). In the present study, we tested the model, using the waveforms of joint torques in walking on level and sloped substrates, and were able to reproduce the discharges of the sensilla without additional tuning of parameter values. These findings support use of the model campaniform sensillum in future studies of sensorimotor control of walking machines.

## METHODS

Experiments were performed on the hind legs of adult female Indian stick insects (*Carausius morosus*, *N* = 14) obtained from a commercial supplier (Backwater Reptiles, United States).

### Sensory Recordings

**Figure 1.**
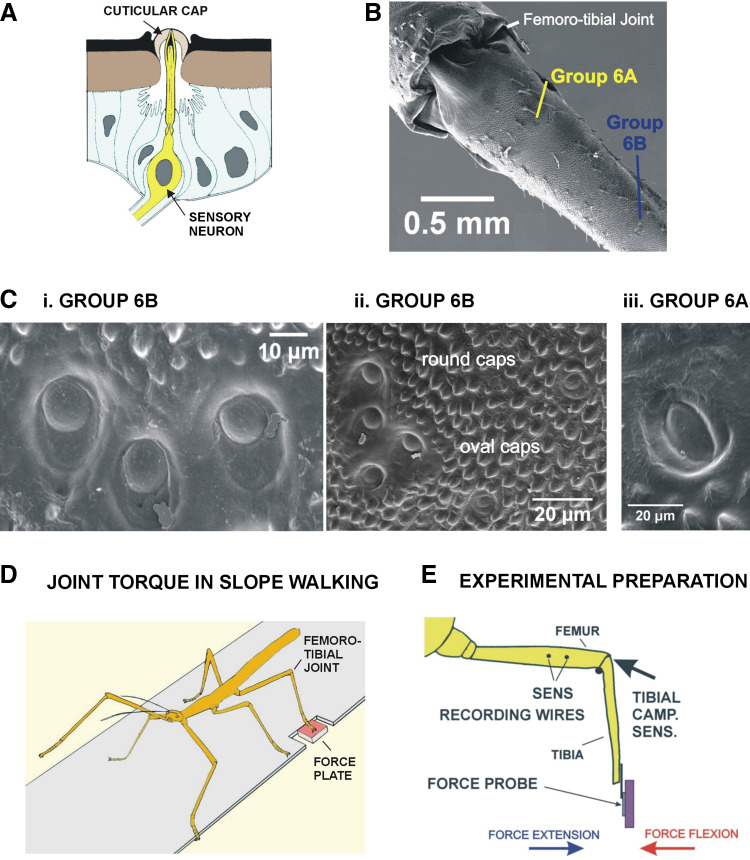
Structure of campaniform sensilla of stick insect hind leg tibia and experimental preparation. *A*: campaniform sensilla monitor forces through a dendrite that inserts to a cuticular cap embedded in the exoskeleton. *B*: scanning electron micrograph of hind leg proximal tibia: the tibial sensilla are arranged in 2 spatially separated subgroups (groups 6A, 6B). *C*: structure of cuticular caps. *i*: The tibial group 6B typically has 3 sensilla with round cuticular caps, similar to the arrangement in middle legs. *ii*: group 6B also contains a variable number of other sensilla that have oval-shaped caps. *iii*: group 6A has 2 sensilla (1 shown) with oval caps oriented close to parallel with the long axis of the tibia. *D*: joint forces (torques) in freely moving stick insects were derived from experiments using a small force plate inserted in a walkway that could be tilted [previous study by Dallmann et al. (13)]. *E*: sensory responses of the tibial campaniform sensilla were recorded in the femur while forces were imposed on the tibia, including ramp and hold waveforms and waveforms of joint torques from freely moving animals.

The techniques used to record activities of stick insect tibial campaniform sensilla (Fig. 1, *A*–*C*) are described in previous publications (23, 25). In the present study, animals were first restrained on a platform and nerves to the left hind leg were severed in the thorax, effectively eliminating all afferent and efferent connections to the central nervous system (CNS). The femur of the hind leg was placed against a small resin block so that the plane of movement of the femoro-tibial joint was in a horizontal plane (Fig. 1, *D* and *E*). Movements at the femoro-tibial joint were eliminated with a pin and a small amount of cyanoacrylate adhesive at the joint. The tarsus was amputated in the distal tibia. For sensory recordings, two 50-μm silver wires (Goodfellow Ltd, AG005825) were inserted through holes made with an insect pin and positioned along the main leg nerve. The insulating layer surrounding the wires was removed over the portion inserted into the leg. Neural activities were monitored during positioning of the wires to achieve proximity to the nerve (nervus cruris) in the femur. The wires were fixed to the cuticle with cyanoacrylate. Sensory activities were recorded with a custom-built amplifier (Michael Duebber, University of Cologne) and stored digitally with a Spike2 interface [Cambridge Electronic Design (CED), Cambridge, UK].

### Mechanical Stimuli

Forces were generated though the Spike2 interface with conventional ramp and hold functions and waveforms of joint torques. The torque waveforms were obtained from a previous study that recorded ground reaction forces of single legs via force plates and three-dimensional kinematics of leg movements via a marker-based motion capture system in freely moving stick insects that walked on level and sloped substrate (Fig. 1*D*; Refs. 13, 23). Torques about the femoro-tibial leg joint were determined by inverse dynamics with a three-dimensional rigid link model. It is important to note that we mimicked joint torques by applying forces on the distal end of the tibia in these tests. However, these sensory responses were consistent with discharges obtained by direct application of forces to the apodeme (tendon) of the tibial flexor muscle (26).

In the present study, the torque values (in the stance phase) were normalized in duration to 800 ms and imported into Spike2 sequencer files and then played and rerecorded with low-pass filtering to eliminate voltage steps. The final output voltages were applied to the tibia with a motor (position controlled; Michael Dübbert, University of Cologne) that displaced a probe containing strain gauges (Fig. 1*E*), producing bending forces on the leg. The torque waveforms of single steps were applied repetitively (mean 0.5 repetitions/s ± 0.004 SD). In this article, a single repetition of the torque waveform is referred to as a “step” or test. Sensory discharge frequencies were stable and showed no long-term adaptation throughout a series of tests.

### Subgroup Identification

**Figure 2.**
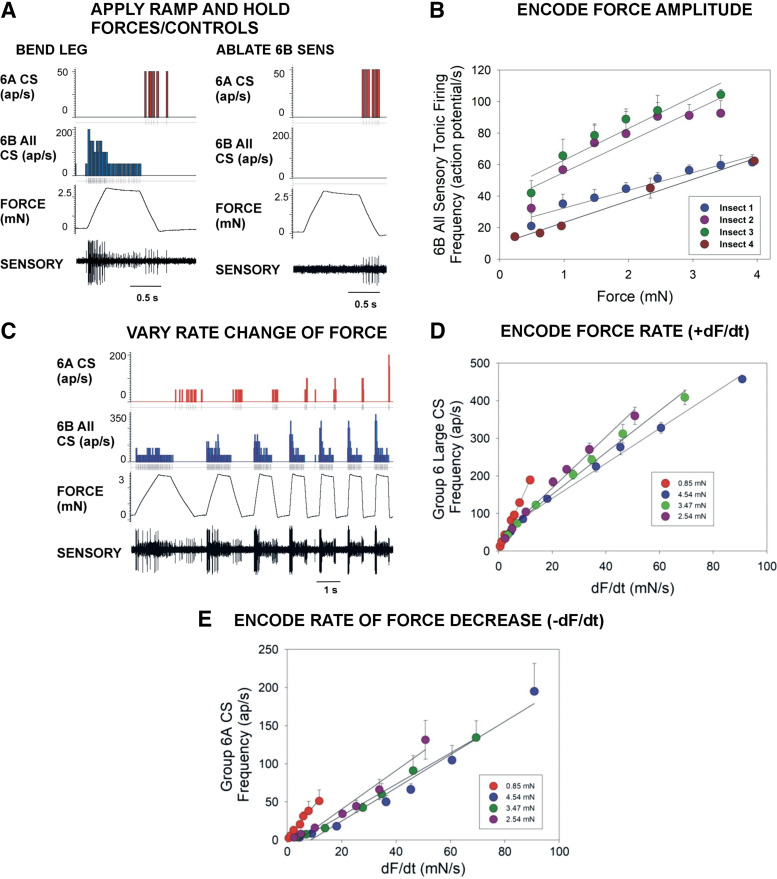
Response properties of tibial campaniform sensilla (CS) of the stick insect hind leg. *A*, *left*: bending forces applied to the distal tibial in the direction of joint extension (movement resisted) elicited vigorous discharges of 6B sensilla during the rising and hold phases and 6A receptors during the ramp decline. *Right*: ablation of 6B receptors selectively eliminated the discharges during the ramp rise and hold. *B*: plot of mean firing frequency of all 6B sensilla during the hold phase to bending forces applied to the distal tibia at different amplitudes in 4 animals. Discharges reflect the force magnitude but vary with stiffness of cuticle. *C*: response to forces applied at different rates of rise and decline. *D* and *E*: plots of mean firing during the ramp rising phase show that 6B large sensilla encode the rate of change of force increase (+dF/d*t*; *D*) and 6A receptors the rate of force decrease (−dF/d*t*; *E*) over a range of force amplitudes. ap, Action potentials. *B*: *N* = 4 animals, *n* = 456 total number of tests. *D* and *E*: *N* = 1 animal, *n* = 217 tests.

Units could be identified by the size of their extracellularly recorded amplitude and time of occurrence within the stimulus. In the present experiments, bending forces were applied to the distal tibia during placement of the recording wires with the goal of maximizing the recorded amplitude of the smallest 6B sensilla. We utilized ablation of the subgroups to confirm unit identification (Fig. 2*A*): after ablation of the caps of 6B sensilla, subsequent retesting to torque waveforms showed that discharges of 6A sensilla were retained because of their proximal location in the tibia (see text below for details). Subsequent ablation of 6A receptors eliminated those components of responses.

### Morphology

Scanning electron micrographs were taken of the isolated tibiae of the hind legs of newly molted animals with a Hitachi S450 microscope (Fig. 1*C*; techniques described in Ref. 26). Light micrographs of caps of campaniform sensilla were obtained from whole mounts of cuticle of the proximal tibia (27).

### Data Storage and Analysis

Data on firing rates and forces were analyzed with Spike2 scripts. The rate of change of force was calculated in Excel. Statistical tests were performed and plotted in SigmaPlot (Systat Software) and SPSS (IBM).

## RESULTS

### Structure and Response Characteristics of Hind Leg Tibial Sensilla in Stick Insects

The tibial campaniform sensilla of the stick insect hind leg occur as two spatially separated subgroups (groups 6A and 6B) on dorsal surface of the proximal end of the tibia (Fig. 1, *A*–*C*), similar to the homologous receptors in the middle leg (25). Group 6A is located ∼0.5 mm and group 6B 1.5 mm distal to the femoro-tibial joint (as measured from the joint condyles). The cuticular caps of the group 6B sensilla are diverse in structure (Fig. 1*C*): there are consistently three sensilla (occasionally 2 sensilla) with caps that appear round (or slightly rectangular), located close to the proximal ends of the surrounding cuticular collars (Fig. 1*Ci*), and a variable number of other receptors with oval-shaped caps in the surrounding (150 μm) cuticle (Fig. 1*Cii*). Group 6A consists of two receptors with ovate caps whose cap long axes are approximately parallel to the tibial long axis, although the posterior sensillum appears tilted ∼20–40° (Fig. 1*Ciii*). The spatial separation of the subgroups greatly facilitated their identification in extracellular recordings, as the caps of the 6B receptors could be ablated (en masse) without affecting firing of 6A receptors. Similar structure and numbers of the tibial sensilla have also been recently reported for the Madagascar stick insect, *Sipyloidea sipylus* (28).

The subgroups of sensilla were differentially activated in tests using bending forces applied to the tibia with ramp and hold functions (Fig. 2) (26, 29). Extracellular recordings consistently (*N* = 12) showed discharges of sensilla of large amplitude during the ramp increases in force in the direction of joint extension that adapted completely (or fired at a low level, <10 Hz) during the hold phase (Fig. 2*A*, *left*). Receptors with smaller spike amplitude fired tonically throughout the hold phases. Ablation of cuticular caps confirmed that these discharges (both larger and smaller spike size) were derived from 6B sensilla (Fig. 2*A*, *right*). Activities of all 6B receptors completely ceased when forces began to decrease, and firing of 6A sensilla occurred when forces declined substantially or approached zero. Tests in which forces were applied to different levels of the hold phase showed that the tonic discharges (sampled late in the hold phase) reflected the force magnitude over a broad range (Fig. 2*B*) but the frequency of sensory discharge depended upon the rate of adaptation, which was prolonged at high force levels (data not shown). Force application at different rates of rise and decline (Fig. 2*C*) indicated that the large sensilla can effectively encode the rate of force increase (Fig. 2*D*) and decrease (Fig. 2*E*), but the discharge frequencies depended upon the force amplitude. These complex features of responses of campaniform sensilla belie their characterization as simple force detectors but are reproduced in the mathematical model of the receptors (see below).

### Forces and Joint Movements in the Hind Leg during Level and Sloped Walking

**Figure 3.**
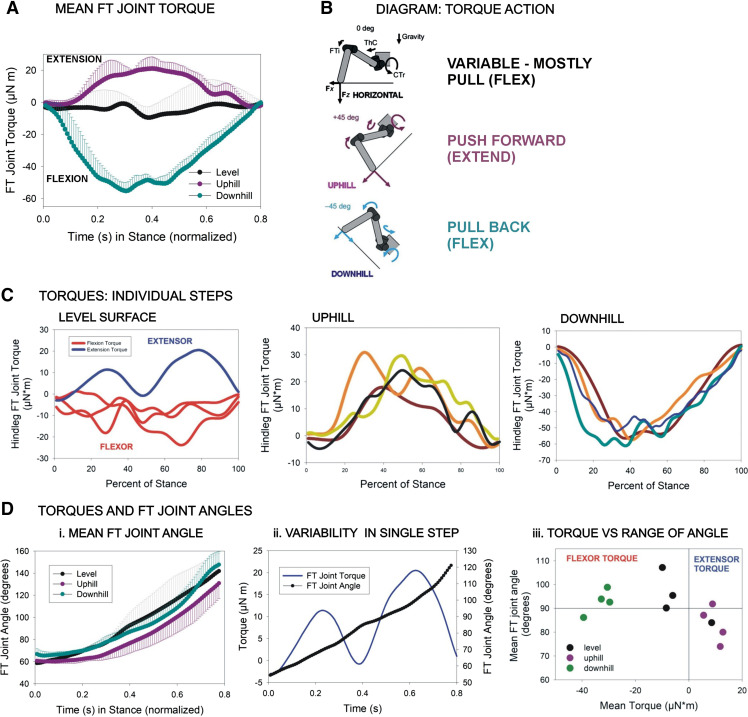
Torques and movements of the femoro-tibial (FT) joint. *A* and *B*: the mean forces (torques) at the FT joint of selected steps used in this study [derived from dataset of Dallmann et al. (13)] vary according to substrate orientation: forces are variable on a level substrate but provide propulsion (extensor torque) in walking uphill and a strong braking force (flexor torque) on downhill slopes. *C*: joint torques of individual steps in walking. The torques of individual steps used in this study showed variability and inflections in walking on level (*left*), uphill (*center*), and downhill (*right*) substrates. *D*: joint movements. *i*: The mean FT joint angle is consistently extended over a relatively constant range during the stance phase of walking on all surfaces. *ii*: Plot of FT joint torque and joint angle in a single sample step. Many individual steps showed substantial variability in joint torques but relatively constant joint movement. *iii*: Plot of the mean joint angles in which torques occurred for steps used in this study (data from Ref. 3). Flexor torques tended to occur in ranges of joint extension, whereas extensor torques were found in ranges of flexion, although torques in walking on horizontal substrates were variable.

The responses of tibial campaniform sensilla were characterized to forces applied to the tibia that were derived (as a subset) from the data of Dallmann et al. (13). That study measured leg movements and ground reaction forces and utilized inverse dynamics to calculate joint torques in freely moving stick insects walking on horizontal and sloped surfaces. In the present study, we utilized both the mean femoro-tibial joint torque as well as selected individual steps. As in our previous study, steps were selected that had average torque values that substantially deviated from the mean [methods of Zill et al. (23)]. We also analyzed the kinematic and kinetic parameters of the selected steps and compared them with all steps in the original data set (13). Figure 3 shows plots of the forces [femoro-tibial (FT) joint torques] and joint movements for the step waveforms used as mechanical stimuli in the present study. The direction and time course of torques at the FT joint in the selected steps generally depended upon the substrate slope (Fig. 3, *A* and *B*): in walking on horizontal surfaces, the torques varied considerably in individual steps (and could change in sign/direction) and the calculated mean torque was quite small in magnitude. In contrast, the mean FT torques were much greater in walking on slopes to oppose the action of the gravitational vector: strong extensor torques were generated in walking uphill that would provide propulsion, whereas large flexor torques were produced to brake forward motion in traversing downhill slopes.

The FT joint torques of individual steps could show substantial variations from the mean (Fig. 3*C*) during the “stance” phase in all substrate inclinations. Torque values were most variable in walking on a level substrate with periods of net extensor or flexor forces (Fig. 3*C*, *left*). Hind leg torques were more consistent in sign in walking on slopes (Fig. 3*C*, *center* and *right*), but many steps had substantial inflections and fluctuations that could reflect adjustments of motor output to forces produced by the placement and lift of the other legs. In contrast, measurements of the FT joint angles in these steps showed much less variability in individual steps, although the ranges of movement varied somewhat (Fig. 3*Di*). Examination of the torque and joint angle values in individual steps showed that, for some steps, small inflections of the values of joint angles could be accompanied by large variations in joint torque (Fig. 3*Dii*). Figure 3*Diii* is a plot of the net torque values (average value in stance) versus the range of joint angles in the steps taken on horizontal and sloped surfaces. This plot shows that in sloped walking flexor torques tended to occur in ranges of joint extension and extension torques in ranges of joint flexion (13). The joint torques could therefore provide support and stability by acting to oppose the effects of body weight on the legs (see discussion).

### Sensory Discharges to Joint Torques Differ in Uphill vs. Downhill Walking and Reflect, but Do Not Simply Encode, the Force Magnitude

**Figure 4.**
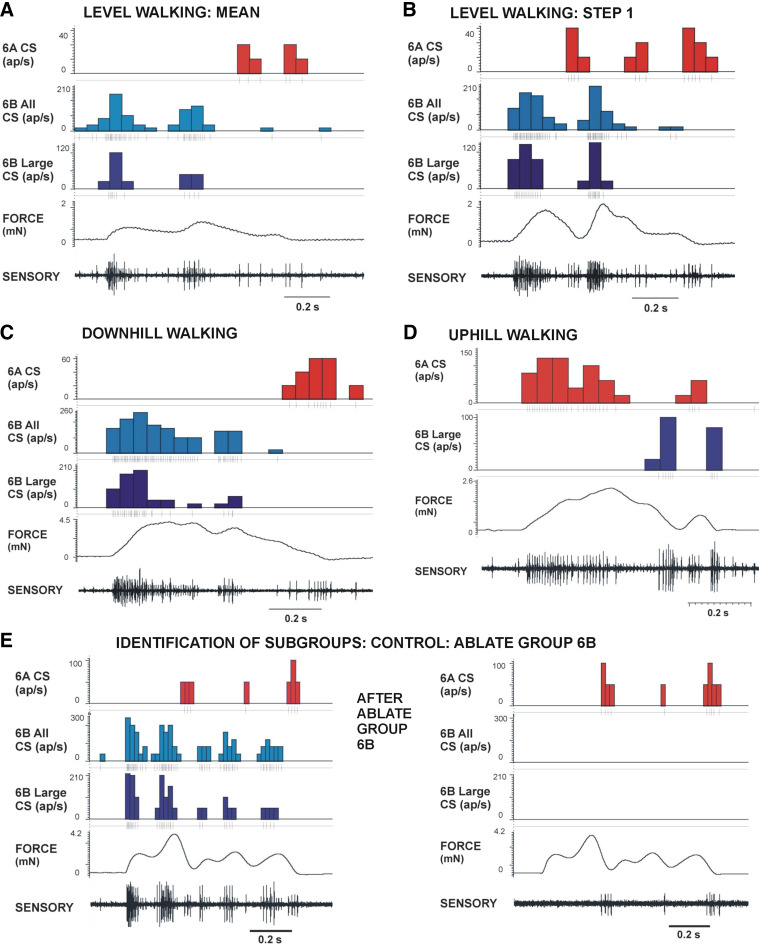
Recordings of sensory discharges to joint torque waveforms. *A* and *B*: walking on level surface. 6B campaniform sensilla (CS) fired in bursts that reflected fluctuations in both the mean forces (*A*) and larger forces that occurred during individual steps (*B*). 6B sensilla discharged during periods of force increase, whereas 6A receptors fired when force decrements declined to low levels. *C*: downhill walking. 6B sensilla fired more intensely during the larger braking forces, but discharges were inhibited when forces fluctuated and briefly decreased even at high sustained levels. *D*: uphill walking. 6A receptors discharged to forces exerted in propulsion, whereas 6B receptors discharged when force decreases approached zero. *E*: controls. Identifications of units derived from subgroups 6B and 6A were based upon action potential size and confirmed by selective ablations of cuticular caps of subgroups. Discharge to force increases (*left*; level walking step) was selectively eliminated by ablation of group 6B (*right*), whereas firing of group 6A receptors persisted after the ablation. ap, Action potentials.

Recordings of activities of tibial sensilla to imposed forces that mimicked walking on slopes showed that the responses of subgroups of sensilla were consistent with the direction of applied force (Fig. 4; all forces shown as positive values). Flexor torques, which were predominant in walking on level surfaces (Fig. 4, *A* and *B*) and larger in downhill walking (Fig. 4*C*), activated 6B sensilla during force increases and could elicit firing of 6A receptors when forces decreased. Extensor torques (consistently occurring in uphill walking) produced the opposite pattern of activation: firing of 6A sensilla to force increases and discharges of 6B receptors to force decrements (Fig. 4*D*).

The sensory discharges were not, however, simply related to the force magnitude, even at high levels of force application. In many steps (Fig. 4, *A*–*D*), firing of large sensilla occurred as a series of bursts and regularly ceased or decreased to very low levels after the maximum force was attained. Discharges of small sensilla could be more sustained and generally reflected the force magnitude (but saturated at high force levels). In addition, small-amplitude sensilla also showed strong hysteresis: firing showed graded increase during force increments, but sensory discharge was completely inhibited when small force decreases occurred during sustained force application, even though the overall force magnitude was high.

### Discharges of Tibial Campaniform Sensilla Are Correlated with Rate of Change of Force in Level and Sloped Walking and Show Hysteresis, Even at Large Force Amplitudes

**Figure 5.**
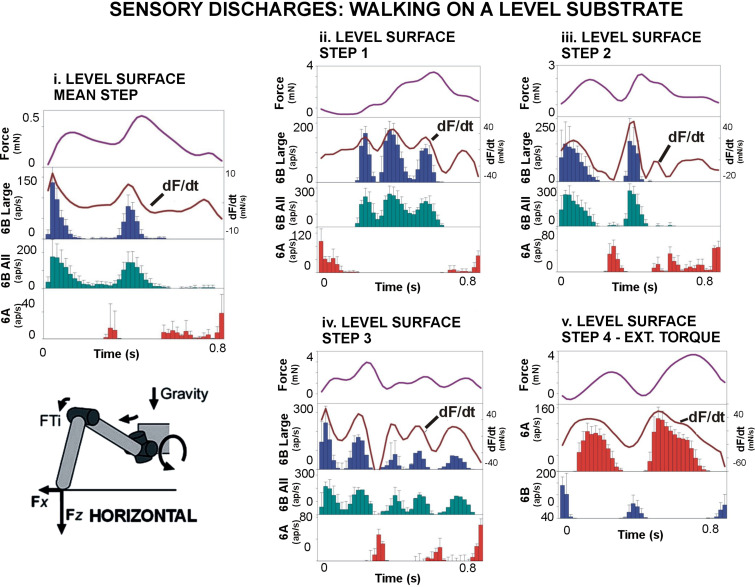
Sensory encoding of joint torques in walking on a level substrate. Plots of pooled data from tests of sensory responses to joint torque waveforms. *i*: Walking on a level substrate. Force levels were relatively small in calculated mean joint torques (*i*) but larger in individual steps (*ii–v*; see scale). In tests with net flexor torques (*i–iv*), 6B sensilla fire to torque increases. The discharge frequency of large 6B sensilla (histogram 2nd trace) and all 6B sensilla (histogram 3rd trace) occurs as bursts that do not follow the force level but instead reflect variations in the rate of change of force (dF/d*t* line overlaid on 2nd trace). In steps with net extensor torques, the 6A sensilla more closely follow the rate of force increases, whereas 6B receptors discharge to force decrements. ap, Action potentials; FTi, femoro-tibial joint; F*x*, forces in direction of walking; F*y*, lateral forces. F*z*, vertical force. Number of tests: *i*: 193, *ii*: 243, *iii*: 216, *iv*: 200, *v*: 200.

Figure 5 shows histogram plots of the sensory discharge rates (mean ± SD) of tibial sensilla to all imposed joint torques in tests of walking on level surfaces, as well as the force magnitude (Fig. 5, *top* trace) and the rate of change of force (+dF/d*t*; line in Fig. 5, second trace). Forces at the FT joint generally occurred as flexor torques that were smaller in magnitude than in slope walking. Both the large- and small-amplitude 6B sensilla discharged in multiple bursts within a step that did not reflect the force level but closely followed the positive values in +dF/d*t* (see Fig. 5, *iii–v*). All afferent activities also showed hysteresis, in that firing ceased (6B large receptors) or sharply declined (6B small receptors) when the rate of change decreased (particularly apparent in Fig. 5, *ii* and *iii*). This could produce a rapid series of discharges within single steps, even when the overall level of force was increasing (Fig. 5*ii*). Activities of 6A sensilla, which discharge to force decreases in ramp and hold tests, only occurred when force levels declined rapidly or to low levels in tests using torque waveforms. These activities were generally at low frequencies, as was observed in tibial sensilla of middle legs (30). Tests of extensor torques in level walking (Fig. 5*v*) produced activation of 6A sensilla, but firing was also limited to the rising phases of force fluctuations and closely followed the changes in the force rate.

**Figure 6.**
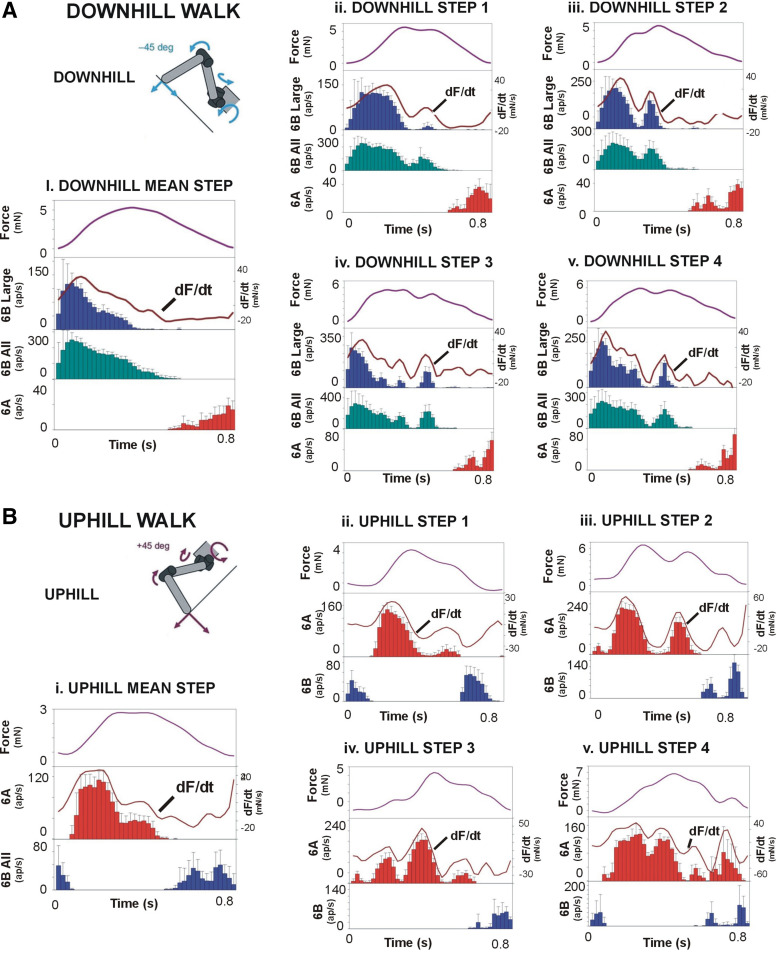
Sensory encoding of joint torques in walking on sloped substrates. *A*: downhill walking. Firing of 6B sensilla reflects the rate of change of force (dF/d*t* overlay line in 2nd trace) and is inhibited by a transient force decrease in a single step (*right*). *B*: uphill walking. 6A receptors fired to force increases when walking uphill. The discharge of the calculated mean torque (*left*) shows considerable adaptation, whereas the firing in an individual step is completely inhibited by a transient force decrease. ap, Action potentials. Number of tests: *A*, *i*: 179, *ii*: 246, *iii*: 216, *iv*: 204, *v*: 206. *B*, *i*: 188, *ii*: 206, *iii*: 225, *iv*: 183, *v*: 195.

Torque values could reach much higher levels in walking on slopes (Fig. 6) and, in tests of downhill walking, could attain twice the values that occurred on level surfaces (compare Fig. 5*iii* and Fig. 6*A*, *iv* and *v*). In these tests, the subgroups of tibial sensilla were activated according to the force direction, as 6B sensilla firing predominated on downhill slopes and 6A signaled positive torques in uphill walking. In downhill walking (Fig. 6*A*), discharges of 6B receptors were more prolonged, particularly in plots including small 6B receptors (Fig. 6*A*, *ii*, *iii*, and *v*). Firing frequencies of small 6B sensilla were modulated but not separated in many discrete short bursts as seen in level walking, in part because the fluctuations in forces were less prominent. However, despite the large force magnitude, the sensory discharges were not sustained during these tests but were largely restricted to the rising phases of force. Also, activities in large- and small-amplitude sensilla showed high sensitivity to variations in the rate of change of force (dF/d*t*) (Fig. 6*A*, *ii* and *iii*) even when these variations produced small changes in force magnitude (rising phase of force in Fig. 6*A*, *iv* and *v*). In addition, afferent firing showed strong hysteresis and often decreased during transient periods in which the rate of change declined, even at high force levels. Discharges of 6A receptors again occurred during large force decreases, and firing was often limited to the period of decline of force at the end of the stance phase.

Discharges of 6A receptors during uphill walking and 6B receptors in downhill walking (Fig. 6) had many of the same characteristics as responses to level surfaces and were largely restricted to the rising phase of the extensor torque (Fig. 6*B, ii* and *iv*). However, the discharge frequencies were extensively modulated and followed the rate of change of force (Fig. 6*Biv*) even when the apparent magnitude of changes in force level were small. In these tests, 6B discharges were restricted to the periods of force decrease but again showed a high sensitivity to the rate of force decrement, and discharges could be initiated by rapid decreases even when the force levels were relatively large.

### Tibial Campaniform Sensilla Show the Same Sensitivity to the Rate of Change of Force in Walking on All Substrate Inclinations

**Figure 7.**
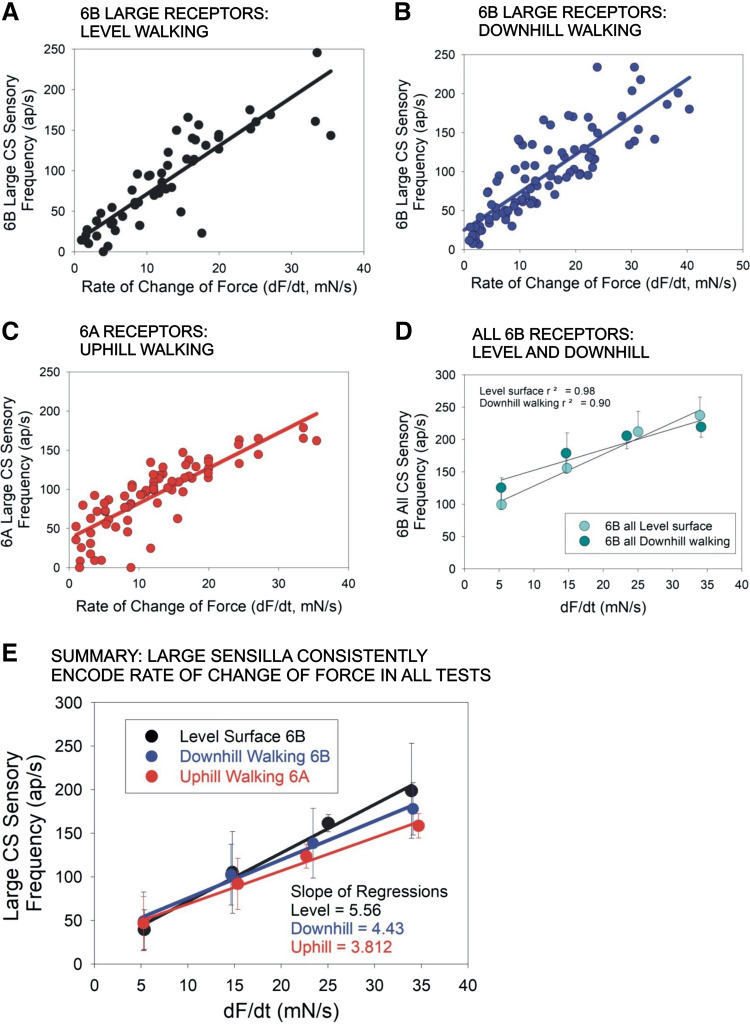
Encoding of rate of change of force (dF/d*t*). *A* and *B*: plots of firing frequencies of large 6B campaniform sensilla (CS) at different dF/d*t* derived from data in Figs. 5 and 6 in walking on level surfaces (*A*) and downhill (*B*). *C*: similar plot of firing of 6A receptors in uphill walking. *D*: pooled, averaged data on mean firing of all 6B sensilla vs. dF/d*t*. *E*: summary plot of firing of 6B large receptors vs. dF/d*t* on substrate orientations. Large 6B sensilla encode the dF/d*t* in all substrate orientations. Animals: *N* = 5. Tests: level *n* = 852 (steps with flexor torques), downhill *n* = 1,051, uphill *n* = 997. ap, Action potentials.

Dynamic sensitivities of campaniform sensilla to joint torques on different substrate slope are compared in the plots in Fig. 7. Figure 7, *A–C*, show plots of the sensillum firing frequencies at different positive rates of change of force (derived and pooled from all steps in the datasets in Figs. 5 and 6) for 6B sensilla in level (Fig. 7*A*) and downhill (Fig. 7*B*) and 6A receptors in uphill (Fig. 7*C*) walking. Although there is some spread in the values of firing frequencies as they depended on both the force level and rate of change, there were general correlations (regression coefficients *R*^2^: Fig. 7*A* = 0.78, Fig. 7*B* = 0.69, Fig. 7*C* = 0.71) that reflect a strong dependence of sensory firing frequency on the rate of force increase. This dependence is also apparent when the data are averaged over ranges of dF/d*t* (Fig. 7, *D* and *E*).

### A Mathematical Model of Campaniform Sensilla Reproduces Sensory Activities in Level and Sloped Walking

We have developed a mathematical model of campaniform sensilla that can successfully reproduce, in software, many characteristics of the sensory transform (input/output) of campaniform sensilla (24). In this model, the biological parameters (response gain, adaptation) are not explicitly determined variables but instead emerge by tuning. For campaniform sensilla, we tuned the model parameters to reproduce the response time courses from tests of responses to the mean joint torques.

**Figure 8.**
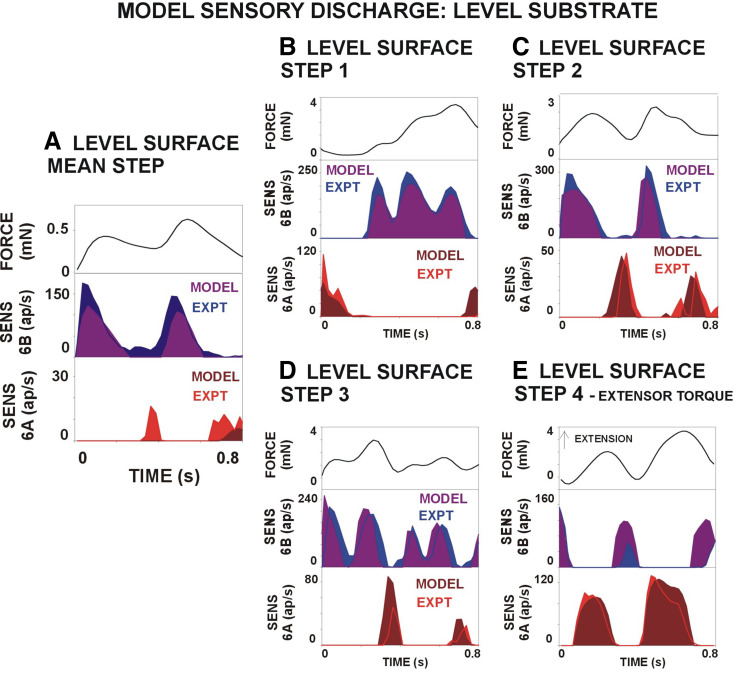
Simulation of sensory discharges in walking on a level surface. Walking on a level substrate, the model produced the same pattern of discharge of 6B receptors to force increases in both the smaller mean torque (*A*) and larger torques of individual steps that varied from the mean (*B–E*). Similar patterns of firing of 6A receptors to force decreases were also seen in simulation to force decreases. ap, Action potentials.

**Figure 9.**
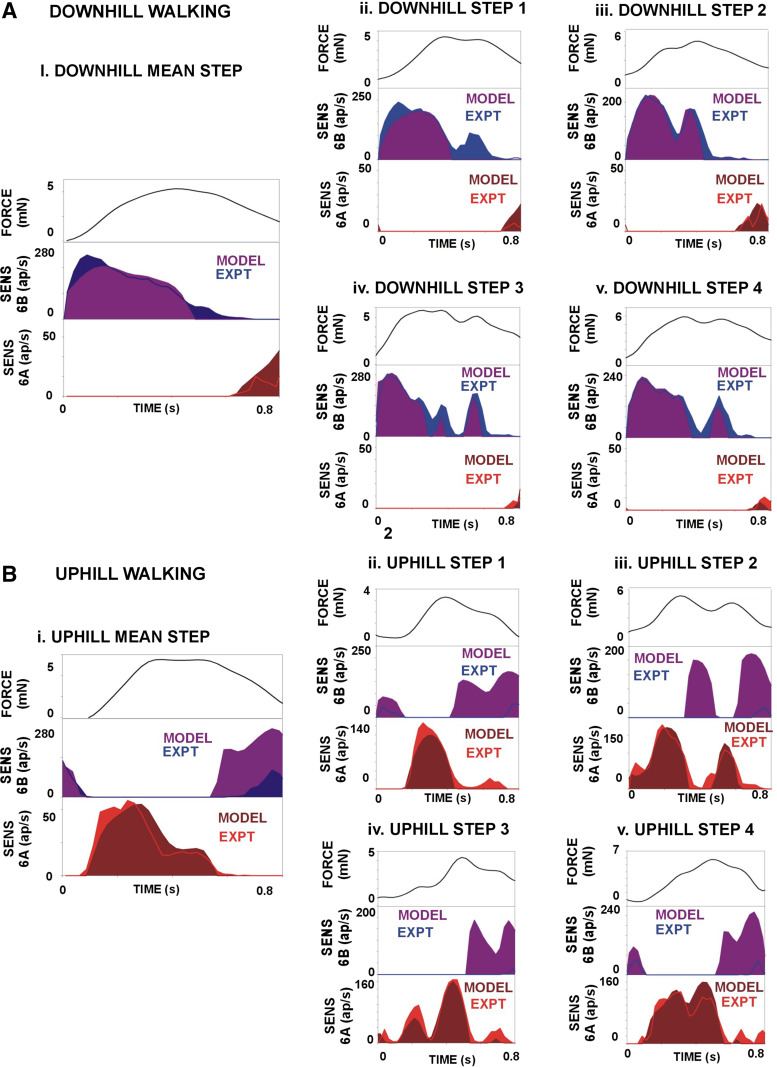
Simulation of sensory encoding of joint torques in walking on sloped substrates. *A*: downhill walking. The model generated firing of 6B sensilla in downhill walking that reflected the rate of change of force. The inhibition that occurred to transient force decrease was also reproduced in some but not all steps. *B*: uphill walking. In simulation, 6A receptors fired to force increases when walking uphill. The discharge of the calculated mean torque (*left*) shows considerable adaptation, whereas the firing in an individual step (*right*) is completely inhibited by transient force decreases. ap, Action potentials.

The model was applied using the force (torque values) from the mean steps in walking on level and sloped surfaces, as well as steps that substantially deviate from the mean [methods of Zill et al. (23)]. Figures 8 and 9 show simulations of the sensory discharges on a level surface (Fig. 8) and on substrates that slope (Fig. 9). The model discharges show many of the same characteristics as the biological sensilla in that *1*) 6B receptors can indicate the torque direction as they are active during force increases in level and downhill walking whereas 6A sensilla fire when walking uphill; *2*) sensilla of opposite subgroups fire during periods of force decrease; and *3*) sensory activity reflects the rate of force development (dF/d*t*) on all substrates. However, the model was less accurate in simulating sensory discharges to force decreases (Fig. 9, *A* and *B*). For example, the mean discharge of 6B sensilla to force decreases in uphill walking was 9.07 (±2.31 SD) action potentials per second, whereas the simulation predicted firing at a higher level (76.2 ± 7.04 spikes/s) (data in Fig. 9*B*).

The model responses share many features with the experimental recordings, despite only being tuned to replicate the response to one stimulus. The other 14 fits are completely “feedforward,” i.e., parameter values are not tweaked to better fit those data. There was a mean absolute error of 10% or less in almost all cases despite no retuning of parameter values, which suggests that the model is capturing fundamental response properties of the tibial sensilla. We observed that tuning the model to each individual trial would improve the fit to that trial (data not shown). This is not surprising, because each cohort of animal subjects would have different properties, e.g., tibia length and cuticle hardness (time from last molt). However, we believe that a model that can reproduce responses of many different animals to widely varying stimuli is most useful for understanding how the nervous system processes force feedback.

## DISCUSSION

Walking on slopes represents a discrete adaptation of behavior in stick insects, as leg movements remain relatively constant while different muscle synergies and levels of activation are generated in countering forces when going uphill versus downhill (13). The present experiments have, for the first time, directly tested responses of force receptors in restrained preparations to joint torques that occur at the femoro-tibial joints in walking of freely moving animals on sloped surfaces. The major findings of this study are that *1*) the subgroups of tibial sensilla are selectively activated in uphill or downhill walking and *2*) the receptors do not simply encode the force magnitude but consistently provide signals about the rate of change (dF/d*t*) of joint torques. In the following we discuss the structure and response properties of the receptors and how the signals about the direction and rate of change of force (dF/d*t*) (termed “yank”; Refs. 31, 32) could aid in adapting walking to achieve stable and relatively constant movements when variations in forces occur because of gait or substrate inclination. These findings are also compared with the results of comparable studies in other animals, including vertebrates.

### Morphology and Response Properties of Hind Leg Tibial Campaniform Sensilla

#### Morphology.

The tibial campaniform sensilla of the hind legs are arranged as two spatially separated subgroups (as in the middle legs; Refs. 26, 27). The group 6A receptors have oval-shaped cuticular caps, typical of stick insect campaniform sensilla (33, 34), whereas group 6B is more complex and has three large sensilla with round cuticular caps in asymmetrical collars and a variable number of other smaller sensilla with oval caps close to (within ∼1.5 mm) the larger receptors. This more dispersed and diverse arrangement is similar to the findings in recent studies of Madagascar stick insects (28) and fruit flies (35, 36), and further studies are planned to quantify these variations. Variability in the number of campaniform sensilla in groups was documented in the original study in the American cockroach of Pringle (37), who reported different numbers of receptors in the right and left legs of the same animal. In the present study, potential differences in afferent responses associated with these smaller receptors were not specifically examined as they often produced very small potentials in extracellular recordings and their caps were difficult to visualize in a dissecting microscope. Identification of units as 6B receptors was based upon ablation of the entire region of the cuticle, and 6A receptors were identified by subsequent more proximal ablations. It is important to note that this variability in morphology may be of limited functional consequence, as campaniform sensilla apparently act postsynaptically in the CNS as populations of receptors (29, 38).

#### Response properties.

Recordings of the tibial sensilla to application of forces as ramp and hold functions showed the same pattern of activities as seen in the middle legs (26, 39): 6B receptors fired during the rising and hold phases, whereas 6A receptors discharged in a separate burst when forces declined (stick insect: Ref. 40; similar results in cockroaches: Refs. 22, 41). Recordings of group 6B similarly showed two classes of sizes of recorded potentials that corresponded to larger more phasic units and smaller tonic units (25). Small sensilla provided much of the sustained discharge during the hold phase and could reflect the force level, although there was individual variability in sensitivity, potentially due to the extent of cuticular sclerotization (Fig. 2*B*). The discharge of the larger units during the ramp rise reflected the rate of change of force (to faster ramps), although the slope of increase also depended upon the stimulus amplitude (Fig. 2*E*). Although the overall discharges of the 6B sensilla reflected force increases over the broad range of force magnitudes that occur in the hind legs, the interactive effects of stimulus amplitude, hysteresis, and rate sensitivity complicate their classification as simple tonic or phasic receptors and afferent encoding was only captured by mathematical modeling (see below).

### Different Subgroups of Receptors Are Activated by Joint Torques in Uphill vs. Downhill Walking

**Figure 10.**
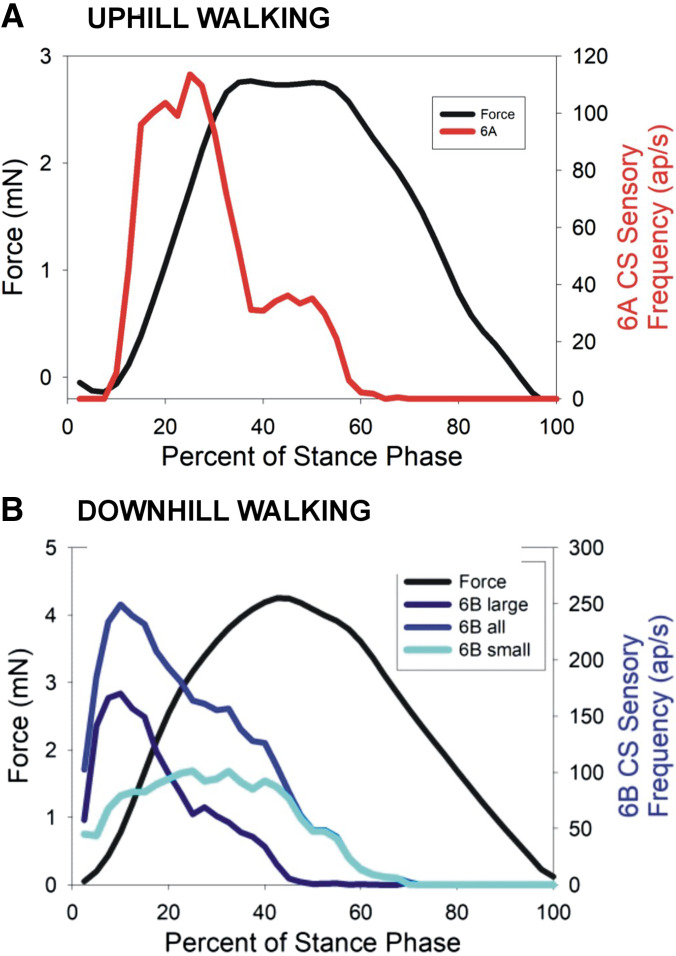
Summary of sensory encoding of joint torques in uphill and downhill walking, including effects of small 6B campaniform sensilla (CS): plots of sensory discharges and mean joint torques in walking uphill (*A*) and downhill (*B*). All sensory discharges are maximal and mostly limited to the rising phase of the force and do not simply reflect the force level, even at high force application. Different subgroups of sensilla are active in walking uphill vs. downhill. The effects of the small 6B receptors (*B*) were calculated by subtracting the firing of large sensilla from the discharges of all sensilla in downhill walking. Small sensillum firing is relatively constant above threshold and provides a signal of sustained force development, although it is limited to the phase of force increase. ap, Action potentials.

All tests showed that different subgroups of tibial campaniform sensilla are activated by joint torques mimicking those occurring in uphill or downhill walking. The discharges of 6A and 6B sensilla to the mean positive torques in walking on different slopes are compared as a summary in Fig. 10. 6A sensilla were activated to the extensor torques that generated propulsive forces when going uphill (Fig. 10*A*), and 6B receptors discharged to flexion torques that produced braking forces in walking downhill (Fig. 10*B*). The pattern of alternating discharge of the subgroups of tibial sensilla is elicited in both cockroaches and stick insects to forces imposed as ramp and hold functions (Refs. 22, 30; Figure 3 in Ref. 39) and is similar to the activities recorded in walking in freely moving or semirestrained animals (25, 41). Thus, the tibial sensilla are effectively acting as proprioceptors in monitoring the torques generated by the animal to adapt to the inclination of the walking surface as well as forces that result from load transfer in gait.

The maximal firing frequencies were obtained in most tests on both uphill and downhill slopes during the initial rise in forces (mimicking the start of stance), which elicited firing of both the larger and smaller group 6B sensilla in level and downhill walking and multiple units of 6A sensilla in going uphill. After the initial increase, either the firing frequencies decreased to a much lower level (Fig. 10*A*) or discharges ceased entirely. These findings reflect the finding that the joint torques occurring at the femoro-tibial joint in walking are largely dynamic in walking of freely moving animals: there is no prolonged hold phase (30, 42). Many torque measurements in walking of vertebrates also never attain a constant level at distal leg joints (2, 43, 44).

### Sensory Discharges in Individual Steps That Deviate from the Mean: Sensilla Encode Positive Values of dF/d*t* but Show Strong Hysteresis

We have found that, in naturally occurring steps that deviated from the mean, sensilla fired in repeated short bursts rather than prolonged discharges, even though the torque magnitude remained large. These bursts were reflective of the *1*) afferent sensitivities to relatively small variations in the rate of force increases and *2*) hysteresis following relatively small decrements in the force rate that could produce large decelerations or complete interruption of the sensory discharges. These characteristics were also seen in discharges of the small campaniform sensilla, which are tonically active to low levels of force. Small sensilla showed more prolonged activity than the large 6B receptors (Fig. 10*B*), but the receptor discharges were inhibited or strongly modulated by small decreases in the force rate. As in our previous study, discharges of sensilla of the opposite subgroup (for example, 6A firing in level and downhill walking) generally did not occur to small force decreases but were at higher threshold (30), although this varied with cuticular properties (stiffness and viscoelasticity; data not shown). All campaniform sensilla at the FT joint are, therefore, generating a fine-grained reflection of the rate of force (torque) increase even in sloped walking when large forces are generated to overcome the effects of gravity.

Our previous study of activities of tibial campaniform sensilla (CS) in the middle legs in walking on a level substrate also found a high sensitivity to dF/d*t* (23). All sensilla directionally encoded the dynamics of force increases and showed hysteresis to transient force decreases. Smaller receptors exhibited more tonic firing. However, the forces applied to the middle legs were of modest amplitude, and it was of interest to see whether the same sensitivities were found when higher forces are generated necessary for sloped walking. The findings of the present study support the idea that dynamic sensitivity in force feedback can modulate ongoing muscle activities to stabilize distal joints even when large forces are generated at proximal joints.

The highly phasic responses that we observed from large tibial receptors parallel the responses observed in other force-detection sensory systems. Sensory discharges that strongly reflect the rate of change of force but do not directly encode the force level were obtained in early studies of bipolar neurons associated with the insertion of the opener muscle in the distal leg segments of crabs (45), similar to the large spike tibial campaniform sensilla of stick insects. A number of studies have also documented sensitivities to the rate of change (dF/d*t*) in vertebrates, in which forces are monitored and controlled as muscle tensions (10, 18, 32, 46–48), as well as invertebrate species, in which forces are detected by cuticular mechanoreceptors or sense organs associated with muscle insertions (45, 49, 50). Although it is not currently known precisely what role dF/d*t* plays in the control of posture and/or locomotion, its ubiquity across a variety of legged animals suggests that it can play an important role in dynamically adapting motor output to the environment (Refs. 32, 51, see also Ref. 52).

### Hysteresis in Force Detection by Campaniform Sensilla

The presence of strong hysteresis in sensory discharges in sloped walking was unexpected. In tests applying forces as ramp and hold waveforms, the effect of the large magnitude of the forces predominated and both the discharges to ramp and hold and mean torque waveforms showed reduced adaptation during the period of force application. However, the steps with torque variations demonstrated that relatively small but rapid decreases could strongly reduce or completely inhibit sensory discharges, even in the small tonic receptors (see Fig. 6, *A*, *iv* and *v*, and *B*, *iv* and *v*). As in our previous study, discharges to force decreases in sensilla of the opposite subgroup (for example, 6A in level and downhill walking) generally did not occur to small force decreases but were elicited by more rapid decreases or by complete unloading at the end of stance (30).

The source of hysteresis in sensory discharges is unknown, although it has been demonstrated in other groups of campaniform sensilla (trochanteral sensilla; Refs. 34, 38, 53) and in receptors that encode kinematic variables (54–57) and is present in sensory processing in the insect nervous system (29, 58). Hysteresis is also seen in discharges of receptors (59) and in sensory encoding in the CNS in vertebrates (60). Golgi tendon organs can also show hysteresis (61), and discharges are completely inhibited by rapid force decreases although very slow decreases produce modulation of firing frequencies (15, 62). Hysteresis may be adaptive and act to reduce residual tensions in leg muscles (50, 63) and compensate for variations in muscle properties (64).

### Functions: Force Sensing at the FT Joint Can Aid in Countering Instabilities

The biomechanical challenge for nervous system control of walking on slopes is to raise or lower the body center of mass while maintaining balance. Our working hypothesis is that the campaniform sensilla at the femoro-tibial joint in the hind legs can aid in meeting this challenge by providing signals that monitor fluctuations in forces and that the CNS can use this information to adjust motor outputs to maintain stability while generating smooth and uninterrupted joint movements. The tests of uphill and downhill walking showed that even though the forces are producing discharges in different subgroups of sensilla, the sensilla are strongly reflecting the same variable (+dF/d*t*) on all substrates (Fig. 7*E*). The tibial sensilla do not directly convey the magnitude of the Newtonian force or reflect the body weight (as, for example, a bathroom weighing scale). Previous studies have provided evidence that the much more numerous CS in groups on the trochanter and proximal femur [coxo-trochanteral (CTr) joint and trochantero-femoral (TrF) joints] provide signals that can strongly affect magnitude and time course of muscle activities that support body load in standing, in the stance phase of walking on a level surface (34, 53, 65), or in climbing an obstacle (66). In contrast, the tibial campaniform sensilla can modulate the muscle activities [set by the central pattern generator (CPG) and sustained by feedback from proximal joints] to tune motor outputs to variations in load. The distribution of forces and torques occurring at different joints has been shown to vary in other motor behaviors such as walking on inverted surfaces or climbing vertical substrates (12), and so there may be flexibility in the force distribution and relative contributions of the CTr and FT groups of sensilla to force outputs in the system (see also Ref. 67). In addition, further understanding of muscle properties would aid in understanding how the patterns of neural activities are transformed to muscle tensions and movements (68).

**Figure 11.**
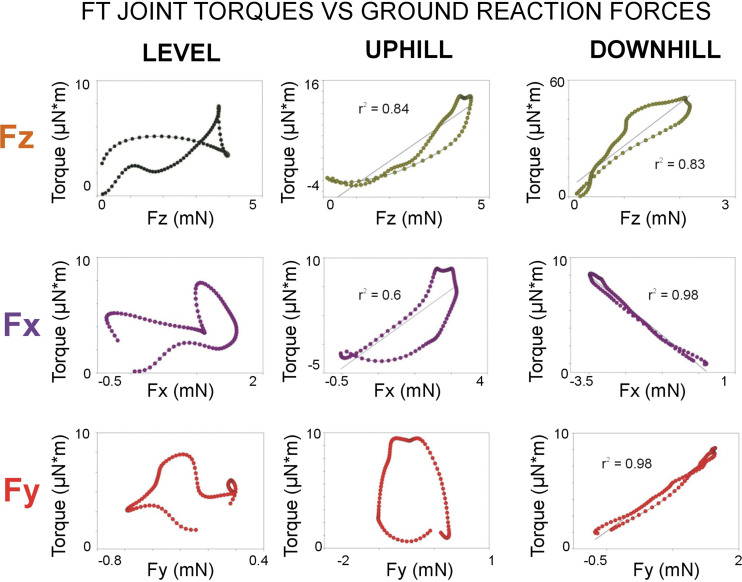
Femoro-tibial (FT) joint torques and ground reaction forces in walking on level and sloped surfaces. These graphs plot the values of the FT joint torques vs. the ground reaction forces during the “stance phase” for all steps (F*x*, direction of walking; F*y*, lateral forces; F*z*, direction of gravity). The torque values are normalized (all positive). See discussion.

Analysis of the relationship between the FT joint torque and ground reaction forces also provides preliminary support for the potential function of tibial CS as corrective or “error”-detecting signals. Figure 11 shows plots of the values of the FT joint torques versus the ground reaction forces during the stance phase for all steps in walking on level and inclined surfaces. Although the plots in walking on a level surface show inconstant relationships, there is a strong correlation of the magnitude of the FT joint torque with ground reaction forces to support body load and counter the effects of gravity in the walking direction (F*x*). In addition, in walking downhill, the FT torques are very strongly correlated with lateral forces (F*y*) that could maintain postural stability when the legs act to pull the tarsus in toward the body (13), as in distributed inward grip (69). In walking downhill, the hind legs are exerting large braking forces, and joint flexor muscles are active even when the joint angle is extending. The effects of gait (lifting of other legs and redistribution of support) could contribute to the large lateral forces in downhill walking and to a high correlation of the FT joint torque with F*y*. This idea is supported by experiments that showed that perturbations of the substrate oriented perpendicular to the body long axis in freely standing cockroaches produce vigorous activation of hind leg tibial campaniform sensilla (22) and motor neurons to leg muscles (Figure 3B in Ref. 22). Further studies that carefully examine movements of multiple legs and ground reaction forces are needed to study the potential effects of gait in producing instability to lateral forces.

### Comparison with Slope Walking in Vertebrates: Most Insects Do Not Have a Vestibular System to Maintain Postural Stability

There are both common features and significant differences in neuromuscular control of walking on slopes in vertebrates and invertebrates. The similarities result from biomechanics and the need to generate additional propulsion in going uphill and braking forces when walking downhill. For example, myographic recordings of muscles at the hip and ankle joints in freely moving cats and humans consistently show enhancement of activities of hip extensor muscles in walking uphill (1, 70, 71), as is seen at the coxo-trochanteral joint in insects (13). Early experiments in humans using perturbations (unloading) imposed on the ankle joint suggested that inputs from receptors that monitor forces (Golgi tendon organs) strongly contribute to these changes (7, 61, 72), although the potential role of sensory signals of force dynamics (18) were not directly evaluated. These effects of force feedback are similar to the functions postulated for the trochanteral campaniform sensilla and coxo-trochanteral muscles in insects (38).

A significant difference is that, whereas the movements of intrinsic leg joints in insects are relatively constant in all substrate orientations, joint movements in vertebrates differ significantly on sloped compared to level substrates (3), particularly at the knee joint (73). Recent studies concluded that control of sloped walking in vertebrates utilizes integration of inputs from both receptors that monitor forces and sensory inputs (muscle spindles) that encode joint position and movement (2, 74, 75). In addition, some of the specific adaptations in walking on slopes in vertebrates may also be related to problems of maintaining postural stability on fewer legs than in insects. Recent analysis of downhill walking in cats suggests that these adaptations result from supraspinal inputs, potentially mediated by the vestibular system, which monitors the effects of gravity and acceleration in vertebrates but is absent in most insects (73). However, the activities of sensory receptors that monitor forces have not, as yet, been studied in vertebrates in walking on slopes (76) to allow comparison with the results obtained from stick insects.

### Are Signals of Dynamic Decreases in Force Preserved in the Stick Insect Central Nervous System?

The present study has shown that when forces are applied using waveforms of joint torques of freely walking animals, signals from the tibial campaniform sensilla strongly reflect the force dynamics (dF/d*t*). It is still unclear how or whether the signals of transient force decreases are encoded in the nervous system. In a previous study we demonstrated that transient increases in forces resisting muscle contraction, signaled in part by the trochanteral campaniform sensilla, can produce increases in activities of motor neurons of stance phase muscles in stick insects (38), but the effects of force decreases were not systematically examined. Campaniform sensilla can potentially have direct effects on motor neurons but have been shown to affect activities in a number of nonspiking interneurons in stick insects (29, 39, 77). These interneurons can act as low-pass filters, but tests using forces applied as ramp and hold functions show that interneurons can reflect both phasic and sustained components of force application, as signaled by campaniform sensilla, but signaling of the rate of change of force were not systematically studied (29). Previous studies have suggested that multimodal proprioceptive feedback can function as “error signals,” as suggested by the present study, but the requisite elements that can differentiate unexpected loads from self-generated forces have not been identified in insects (76). Further studies are necessary to understand how force dynamics can be preserved in motor outputs during “active” behaviors, such as walking (78).

### Discussion of Mathematical Model

These tests show that the model we have developed, although tuned with limited datasets, can describe a variety of additional responses with these same parameter values, suggesting that the model replicates the underlying dynamics of CS afferents without overfitting to the data, although further tuning of the model might improve predictions of discharges to decreasing forces. These findings also demonstrate that similar parameters are encoded in walking uphill and downhill, supporting the hypothesis that insects do not use different motor programs in sloped walking (13) but utilize the same local mechanisms of motor control on all substrates.

### Limitations of This Study

The waveforms of joint torques of the hind leg FT joint that were used as mechanical stimuli in this study were derived by inverse dynamics from a previous study of sloped walking of free-moving animals (Ref. 13; see also Refs. 23, 79, 80). The method of inverse dynamics makes the assumptions that limbs are composed of rigid segments and leg joints are frictionless (81, 82). In the study of Dallmann et al. (13), the joint torques at the most proximal leg joint (body-coxa) were closely correlated with recordings of muscle activities, supporting the validity of the method. It is also important to note that, in the present study, many of the characteristics of afferent discharges, such the sensitivities to small force decreases, were evident in the discharges of sense organs to all waveforms, despite the variations of their detailed dynamics or amplitude. In addition, the waveforms were applied to the leg without control for the effects of viscoelasticity of the cuticle. However, we found that in using the dynamic waveforms applied in the present study, the resultant forces closely (within 10%) followed the shape of the torque waveforms and the effects of viscoelasticity, such as stress relaxation, were minimal.

### Conclusions and Future Work

This study has recorded the activities of sense organs that monitor forces and shown that feedback during walking can provide specific information that could aid in countering instabilities in traversing slopes. The findings also suggest that force feedback and information about force dynamics are not monotonic but may be related to the specific functions of individual leg joints. Succeeding experiments should examine responses of the trochanteral and femoral campaniform sensilla (the largest groups of receptors) to torque waveforms to test whether the dynamic sensitivities and hysteresis are similar to those found in the tibial sensilla in the present study. These characteristics of force detection may also be beneficial in the control of legs of walking machines in locomotion on sloped surfaces.

## DATA AVAILABILITY

Data will be made available upon reasonable request.

## GRANTS

This study was supported by National Science Foundation (NSF Collaborative Research in Computational Neuroscience) CRCNS Grant 2113028.

## DISCLOSURES

No conflicts of interest, financial or otherwise, are declared by the authors.

## AUTHOR CONTRIBUTIONS

S.N.Z., C.J.D., and N.S.S. conceived and designed research; S.N.Z. and W.Z. performed experiments; S.N.Z., C.J.D., W.Z., H.C., and N.S.S. analyzed data; S.N.Z., C.J.D., W.Z., H.C., C.G., and N.S.S. interpreted results of experiments; S.N.Z., C.J.D., W.Z., and N.S.S. prepared figures; S.N.Z. and N.S.S. drafted manuscript; S.N.Z., C.J.D., W.Z., C.G., and N.S.S. edited and revised manuscript; S.N.Z., C.J.D., and W.Z. approved final version of manuscript.
